# Asymptomatic Patients with Novel Coronavirus Disease (COVID-19)

**DOI:** 10.4274/balkanmedj.galenos.2020.2020.4.20

**Published:** 2020-06-01

**Authors:** Peng An, Ping Song, Yong Wang, Bo Liu

**Affiliations:** 1 Department of Radiology, Xiangyang Xiangyang No. 1 People’s Hospital Affiliated to Hubei University of Medicine, Xiangyang, China; 2 Department of Infectious Diseases, Xiangyang No. 1 People’s Hospital Affiliated to Hubei University of Medicine, Xiangyang, China; 3 Department of Public Health, Xiangyang No. 1 People’s Hospital Affiliated to Hubei University of Medicine, Xiangyang, Hubei, China; 4 Department of Respiratory and Critical Care Discipline, Xiangyang No. 1 People’s Hospital Affiliated to Hubei University of Medicine, Xiangyang, China; # An and Song contributed equally to this work.

To the Editor,

Since the outbreak of the new coronavirus in Wuhan in December 2019, it has now spread to more than 100 countries around the world. The cumulative number of confirmed cases is as high as 1.1 million, with 58,000 deaths, bringing huge challenges to the people world-wide. The Chinese government has curbed the rising epidemic situation in China through strict prevention and disease control mechanisms (1,2,3). At present, there are only 1,021 confirmed cases and only 300 critical cases (4). Hubei Province, the epicenter of the epidemic, has been deregulated, whereas Wuhan will be deregulated on April 8. However, while celebrating the victory over pneumonia, we should not ignore a very serious challenge. There may be many asymptomatic patients in China. As yet, there are 1,400 known asymptomatic patients in China (4). Since many asymptomatic patients are unknown and therefore not isolated, it is very difficult to control the outbreak. Therefore, in this study, we present 25 asymptomatic patients that we hope will give significant insights into COVID-19. This work was approved by the ethics committee of Xiangyang first people's Hospital Affiliated to Hubei University of Medicine [(2019) LSZ (S109)] and informed consent was obtained from the patients for clinical research.

Of these 25 patients, 22 were family members who took care of confirmed patients with COVID-19. The other three patients were staff involved in cleaning of medical waste and in transportation in the hospital. Two out of the three cases were men. Most of these cases were young patients with an average age of 42 years. An interesting finding was that most of the asymptomatic patients had blood type O. Table 1 provides a detailed description of the patients’ characteristics.

Table 2 shows the characteristics of chest computed tomography (CT) scans of asymptomatic patients. In total, 24 of these 25 patients had abnormal CT findings in the lung. Approximately two-thirds of the patients had an involvement of a single lobe, and two-thirds had only a ground-glass density shadow. The least common CT finding was interlobular septal thickening (Figure 1). All the patients were isolated after diagnosis. In total, 16 of them recovered without any symptoms. However, nine patients developed mild cough and/or other symptoms. These nine patients received medication (Chloroquine phosphate, 500 mg twice daily for seven days and Abidol, 200 mg three times a day for no more than ten days) and their symptoms improved over time.

Control chest CT was performed in all patients 7-30 days after isolation. It was observed that there was improvement in lesions and their involvement had decreased in 24 patients. None of them had further radiological progress. In the patient who did not have any radiological findings at the time of diagnosis, no radiological findings were observed on the follow-up CT.

According to the “Pneumonia Diagnosis and Treatment Program for New Coronavirus Infection (Trial Seventh Edition)” issued by the National Health Commission, the diagnosis of COVID-19 pneumonia is mainly based on the comprehensive judgment of epidemiological history, clinical manifestations, imaging examination, and detection of new coronavirus nucleic acid. Although nucleic acid detection is the gold standard for the diagnosis of COVID-19, false negative test results are not too low because of many factors such as test kit, sampling tube, specimen sampling, drugs administered to patients etc. (5).

Chest CT is of great value in the diagnosis of COVID-19 because of its simplicity and ease, especially in patients with or without clinical symptoms (3,6,7). Chest CT examination is particularly important because it is conducive to the early detection of asymptomatic infections, hence resulting in early isolation of the positive cases and reduction of infections in people in contact (8).

In this study, we observed that one-third of the asymptomatic patients with COVID-19 developed symptoms at follow-up. These asymptomatic patients are usually young and have an excellent prognosis. More than 90% of these asymptomatic patients have radiological abnormal findings in diagnosis. These pulmonary lesions are mostly below the peripheral pleura of the lower lobes and are characterized by ground-glass density shadow.

As a result, chest CT plays an important role in the early diagnosis of COVID-19 and leads to the early isolation and treatment of asymptomatic patients. We believe that this process is vital in controlling the outbreak.

## Figures and Tables

**Table 1:**
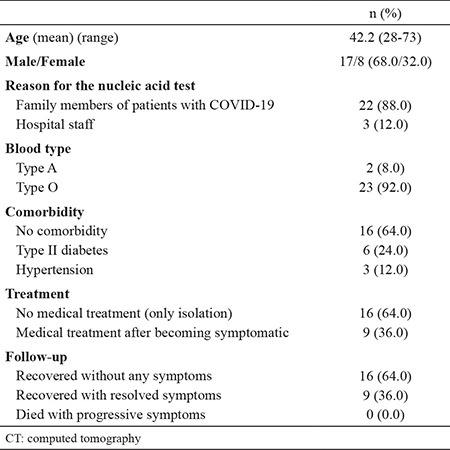
Characteristics of asymptomatic patients with positive nucleic acid test for Covid-19

**Table 2:**
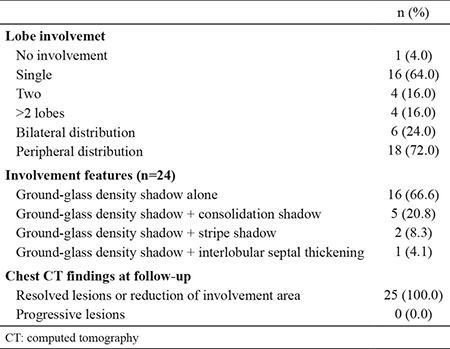
Chest CT Charecteristics of asymptomatic patients

**Figure 1:**
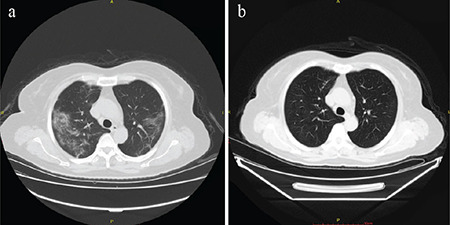
A 59-year-old man presented to the hospital because of close contact with a patient with COVID-19. Chest CT at admission shows multiple ground-glass shadows with interlobular septal thickening (a). After 14 days of isolation, chest CT results were normal (b). The patient recovered and was then discharged from the hospital. CT: computed tomography
